# Value-based health care in heart failure: Quality of life and cost analysis

**DOI:** 10.1016/j.clinsp.2023.100294

**Published:** 2023-10-29

**Authors:** Eduarda Chiesa Ghisleni, Vitória Rech Astolfi, Larissa Zimmermann, Camila Nogueira Leandro Lira, Eduarda Faria do Nascimento, Ana Paula Beck da Silva Etges, Fabiana G. Marcondes-Braga, Fernando Bacal, Luiz Claudio Danzmann, Carisi Anne Polanczyk, Andreia Biolo

**Affiliations:** a Faculdade de Medicina, Universidade Federal do Rio Grande do Sul, Porto Alegre, RS, Brazil; b Post-Graduate Program in Cardiology, Universidade Federal do Rio Grande do Sul, Porto Alegre, RS, Brazil; c Hospital de Clínicas de Porto Alegre, Porto Alegre, RS, Brazil; d Faculdade de Medicina, Universidade Luterana do Brasil, Canoas, RS, Brazil; e Hospital Universitário de Canoas, Canoas, RS, Brazil; f Instituto do Coração do Hospital das Clínicas da Faculdade de Medicina da Universidade de São Paulo, São Paulo, SP, Brazil; g National Institute of Science and Technology for Health Technology Assessment (IATS) ‒ CNPq/Brazil (project: 465518/2014-1), RS, Brazil; h Faculdade de Tecnologia, Pontifícia Universidade Católica do Rio Grande do Sul, Porto Alegre, RS, Brazil; i Post-Graduation Program in Epidemiology, Faculdade de Medicina, Universidade Federal do Rio Grande do Sul, Porto Alegre, RS, Brazil; j Hospital Israelita Albert Einstein, São Paulo, SP, Brazil; k Hospital Moinhos de Vento, Porto Alegre, RS, Brazil

**Keywords:** Heart failure, Value-based health care, Quality of life, Patient reported outcomes measures, Costs in health

## Abstract

**Objectives:**

To measure Quality of Life (QoL) and costs of Heart Failure (HF) outpatients in Brazil as an introduction to the Value-Based Health Care (VBHC) concept.

**Materials and methods:**

Cross-sectional study, patients with HF, with ejection fraction <50%, were recruited from three hospitals in Brazil. Two QoL (36-Item Short Form Survey [SF-36] and Minnesota Living with Heart Failure Questionnaire [MLHFQ]) and two anxiety/depression questionnaires were applied. SF-36 scores were stratified by domains. Treatment costs were calculated using the Time-Driven Activity-Based Costing (TDABC) method. Results were stratified by NYHA functional class and sex.

**Results:**

From October 2018 to January 2021, 198 patients were recruited, and the median MLHFQ (49.5 [IQR 21.0, 69.0]) and SF-36 scores demonstrated poor QoL, worse at higher NYHA classes. A third of patients had moderate/severe depression and anxiety symptoms, and women had higher anxiety scores. Mean costs of outpatient follow-up were US$ 215 ± 238 for NYHA I patients, US$ 296 ± 399 for NYHA II and US$ 667 ± 1012 for NYHA III/IV. Lab/exam costs represented 30% of the costs in NYHA I, and 74% in NYHA III/IV (US$ 63.26 vs. US$ 491.05).

**Conclusion:**

Patients with HF in Brazil have poor QoL and high treatment costs; both worsen as the NYHA classification increases. It seems that HF has a greater impact on the mental health of women. Costs increase mostly related to lab/exams. Accurate and crossed information about QoL and costs is essential to drive care and reimbursement strategies based on value.

## Highlights

• Value-based models might improve care for heart failure.
• The authors observed poor QoL and high treatment costs in HF outpatients in Brazil.
• Women seem to have worse quality of life, more anxiety and depression symptoms.

## Introduction

Heart Failure (HF) has become a prevalent and costly disease for countries, along with health care costs that reached almost 18% of the United States GPD in 2019.^1^ Additionally, HF is an important cause of mortality and morbidity, resulting in worse Quality of Life (QoL) than other diseases.^2^ Despite the emergence of new therapies, the quality of care is below expectations to improve outcomes.^3^, ^4^, ^5^

Value-Based Health Care (VBHC) is a proposal for restructuring healthcare systems, aiming to improve the quality of service delivered by considering what matters to the patient and reducing costs for the system.^6^ The measurement of mortality and hospitalization is important in HF; nevertheless, overall wellness and daily activities have turned out to be as important over the lifetime. For that, health systems should be organized to cover the full cycle of care to achieve better outcomes,^7^ requiring significant changes in health systems, including bundled payments, standardized outcome measures, and technological advancement.^8^

Patient Reported Outcomes Measurements (PROMs) are the standardized outcome measurement method proposed by the International Consortium for Health Outcomes Measurement (ICHOM),^9^ which allows the evaluation of the quality of the care services and current patients’ health status. The information collected by PROMs may improve care and identify disease-aggravating factors.^10^^,^^11^

Most current value-based models in cardiology focus on payment reforms that encourage quality of service.^12^ Several other factors must be considered, such as frailty, educational level and QoL, which are also associated with health expenses.^13^^,^^14^ To address this question, the objective of this study is to analyze current Brazilian care for HF outpatients. Here, the authors describe QoL, anxiety, depression metrics, and costs stratified by NYHA functional classification of outpatients with HF. The authors believe that these are essential steps to improve the quality of care and move toward VBHC.

## Materials and methods

This was a multicentric, cross-sectional study conducted from October 2018 to January 2021. Three hospitals in Brazil (from the south and southeastern regions) participated in the study: Hospital de Clínicas de Porto Alegre (Hospital 1), Instituto do Coração (Hospital 2), and Hospital Universitário de Canoas (Hospital 3).

All the participants' hospitals are national reference centers for HF, which contributes to the similar clinical characteristics (advanced disease) in our sample of patients. While Hospitals 1 and 3 are references in the south of Brazil, Hospital 2 is a reference in the southeastern and from distant regions such as the north. Hospital 3 has the smallest clinic of the centers.

### Sample of patients

Inclusion criteria were patients with HF diagnosed for more than 6 months, Left Ventricular Ejection Fraction (LVEF) lower than 50% documented for less than 12 months, and a signed Free and Clarified Consent Term (FCCT). Once a week, patients who had an outpatient appointment in the following days and met the inclusion criteria were invited to enter the study; they were also free to refuse participation. Exclusion criteria were chronic diseases that could have made it difficult to assess HF symptoms (e.g., chronic obstructive pulmonary disease with important dyspnea and conditions that limited movement such as morbid obesity or advanced osteoarthritis) and negative FCCT assignment.

The study was approved by each hospital's Research Ethics Committee (CAAE 89062818.5.1001.5327) and was performed in accordance with the national ethical and research regulations. STROBE recommendations were followed.

### Quality of life assessment

Two validated questionnaires were used for QoL assessment, and the other two were applied for detecting symptoms of anxiety and depression. The first was the 36-item Short Form Health Survey (SF-36),^15^ which is a generic QoL assessment questionnaire and evaluates eight aspects (Physical Functioning; Role Physical; Body Pain; General Health; Vitality; Social Functioning; Role Emotional; Mental Health). The results were transformed into a scale of 0–100, in which zero was considered the worst and 100 was the best QoL score. The second one is a specific questionnaire for HF, the Minnesota Living with HF Questionnaire (MLHFQ),^16^ a 21-item instrument about physical, socioeconomic, and emotional aspects of HF based on patients’ perceptions. It is assessed using a score of 0‒105 (< 24 represents good quality of life, 24‒45 moderate quality and > 45, represents poor quality of life).

The authors also used Beck Anxiety and Depression Inventory (BAI and BDI),^17^^,^^18^ which are questionnaires developed to detect symptoms, although they are not capable of making a diagnosis. Both are self-reported; BAI is rated 0‒63 (0‒21 means low anxiety, 22‒35 moderate levels of anxiety and > 35 potentially concerning levels of anxiety) and composed of 21 questions. The BDI also ranges from 0‒63 (0‒9 minimal depression; 10‒18 mild depression; 19‒29 moderate depression; and > 29 severe depression) and is composed of 21 questions.

In all centers, questionnaires were administered in a private room. At the main center, the team was trained by a psychiatrist who was not part of the recruitment team; orientations were then replicated for the other centers. One professional from each center received orientation and taught the others; they were instructed to all act the same way to reduce bias. The orientations are specified in Appendix 1. Lab tests and clinical history were revised in the patient's electronic medical records.

### Cost assessment

The Time-Driven Activity-Based Costing (TDABC) method was used to guide the cost assessment. The TDABC is recognized as the gold standard method of performing micro-costing studies in health care, taking into account the patient's full cycle of care.^19^^,^^20^ The principle of estimating costs is to measure the time and the type of labor and nonlabor resources consumed per patient.^21^

For the method's application, the eight TDABC steps were followed, reaching a total cost per $/h for each patient.^22^ Salaries were estimated in each center, according to information acquired in the financial sector, except in Hospital 2, which did not agree to share information, so cost information from Hospital 1 was used as a reference. An expense analysis was carried out about patients’ transportation, costs of medication, number of visits (physician/nurse/nutritionist), and labs/exams in a 6-month period after the interview. For medication cost standardization, the National Health Price Bank (BPS 2019) was used.

Costs of infrastructure, laboratory, and exams (including imaging) from each patient were computed with Hospital 1 as a reference. For time measurement, a health professional took note of the length of, at least, three medical, nurse and nutritionist appointments to calculate an average in each center.

### Statistical analysis

Demographic characteristics were stratified according to center, and QoL and costs were analyzed according to NYHA functional classification or sex.

Analyses were performed using R Statistical Software RStudio (v1.1.456, RStudio Team (2020) RStudio: Integrated Development for R. RStudio, PBC, Boston, MA). Continuous variables were evaluated for distribution symmetry by histograms and were expressed by median and interquartile range or mean and standard deviation. For continuous asymmetrical distributions/nonnormal variables, the Mann-Whitney test was used to compare differences, and for symmetrical distributions/normal variables, Student's *t*-test was used. Differences in qualitative variables were compared by the chi-square test, with Yates continuity correction, when necessary. Additionally, multiple comparisons were corrected by the Bonferroni method. To make correlations between test scores, Spearman's correlations were employed, and multiple comparisons were corrected by the Holm-Bonferroni method in the correlation plots.

To estimate the effect of NYHA classification on quality of life (measured by MLHFQ), the authors used a multivariable linear regression model, choosing potential confounders through an extensive literature review and a cutoff of 0.2 on bivariate analysis. Each variable was added in steps and in accordance with the proposal of Mickey et al.^23^ Collinearity was evaluated by Variation Inflation Factors (VIFs), and residual analysis was checked for homoscedasticity and normality. The model with the best explanatory capacity was chosen by adjusted R-squared.

Cost results were reported as the mean (SD ‒ Standard Deviation) or median (IQR I Interquartile Range) and stratified by NYHA functional classification. Cost data were collected and analyzed in Brazilian currency and reported in Reais (R$ in 2020) and international dollars according to the Purchasing Power Parity (PPP) value (2.362 in 2020). A radar chart tool from Microsoft Excel (Microsoft Corporation, Redmond, WA) was used to visually display QoL scores and costs stratified by NYHA functional classification or sex.

All analyses considered an alpha of 0.05 and/or confidence interval limit containing the unit.

## Results

A total of 198 patients were included, 112 from the primary center (Hospital 1), 48 from Hospital 2, and 38 from Hospital 3. Of the whole sample, 56% were men, the median age was 58 years old [48.2, 67.0], the median LVEF was 29% [24.0, 35.7], ischemic cardiomyopathy corresponded to 26.3% of the HF etiology, and the functional classification was as follows: 28.3% NYHA I, 46.5% NYHA II and 25.3% NYHA III/IV. All patients were ACC/AHA stage C HF. Seventy-six percent of the patients were in use of angiotensin-converting enzyme, angiotensin receptor blocker, or angiotensin receptor-neprilysin inhibitor, 87% were in use of beta-blocker and 52% in use of aldosterone antagonist. A higher percentage of patients from Hospital 2 were in use of hydralazine/nitrate due to kidney disease. Also, there were more patients with Chagas disease cardiomyopathy in this hospital due to demographic issues. Most of the sample had a low educational level, and the median monthly income was $ 221.25 (183.80, 384.40). The demographic characteristics among the centers were very similar despite the difference in the number of patients (Table 1).

**Table 1:** Characteristics of patients stratified by center.

	Overall	Hospital 1	Hospital 2	Hospital 3
**N**	198	112	48	38
**Male sex**	110 (55.8)	63 (56.3)	27 (56.2)	20 (52.6)
**Age (Years) – median [IQR]**	58 [48.2, 67.0]	61.0 [52.7, 68.2]	50.0 [36.0, 58.0]	61.0 [54.5, 71.0]
**Finished School**	67 (33.8)	33 (29.5)	25 (52.1)	9 (23.7)
**Income (US$) – median [IQR]**	226.10 [187.91, 392.83]	228.00 [187.53, 403.75]	213.28 [198.55, 380.00]	209.00 [187.06, 380.00]
**Clinical features of HF**				
LVEF ‒ % [IQR]	29.0 [24.0, 35.7]	28.0 [24.0, 34.0]	29.5 [23.0, 36.0]	33.5 [27.0, 38.7]
Ischemic cardiomyopathy	52 (26.3)	30 (26.8)	8 (16.7)	14 (36.8)
Hypertensive cardiomyopathy	23 (11.6)	17 (15.2)	3 (6.2)	3 (7.9)
Chagas’ cardiomyopathy	11 (5.5)	2 (1.8)	9 (18.8)	0 (0.0)
**NYHA Functional Classification**				
I	56 (28.3)	38 (33.9)	10 (20.8)	8 (21.1)
II	92 (46.5)	51 (45.5)	21 (43.8)	20 (52.6)
III/IV	50 (25.3)	23 (20.5)	17 (35.4)	10 (26.3)
**Medical History**				
Hypertension	138 (69.7)	91 (81.2)	18 (37.5)	29 (76.3)
Diabetes	65 (32.8)	39 (34.8)	10 (20.8)	16 (42.1)
Atrial fibrillation	39 (19.7)	21 (18.8)	11 (22.9)	7 (18.4)
Smoking	89 (45.4)	55 (49.5)	14 (29.2)	20 (54.1)
**Prescription**				
ACE inhibitor/ARB/ARNI	151 (76.3)	95 (84.8)	36 (75.0)	20 (52.6)
Betablocker	173 (87.4)	110 (98.2)	39 (81.2)	24 (63.2)
Aldosterone antagonist	103 (52.0)	51 (45.5)	37 (77.1)	15 (39.5)

Variable distributions are reported as n (%) unless otherwise specified. IQR means Interquartile Range.

ACE, Angiotensin Converting Enzyme; ARB, Angiotensin Receptor Blocker; ARNI, Angiotensin Receptor-Neprilysin Inhibitor; LVEF, Left Ventricular Ejection Fraction; NYHA, New York Heart Association.

### Quality of life

**Fig. 1:**
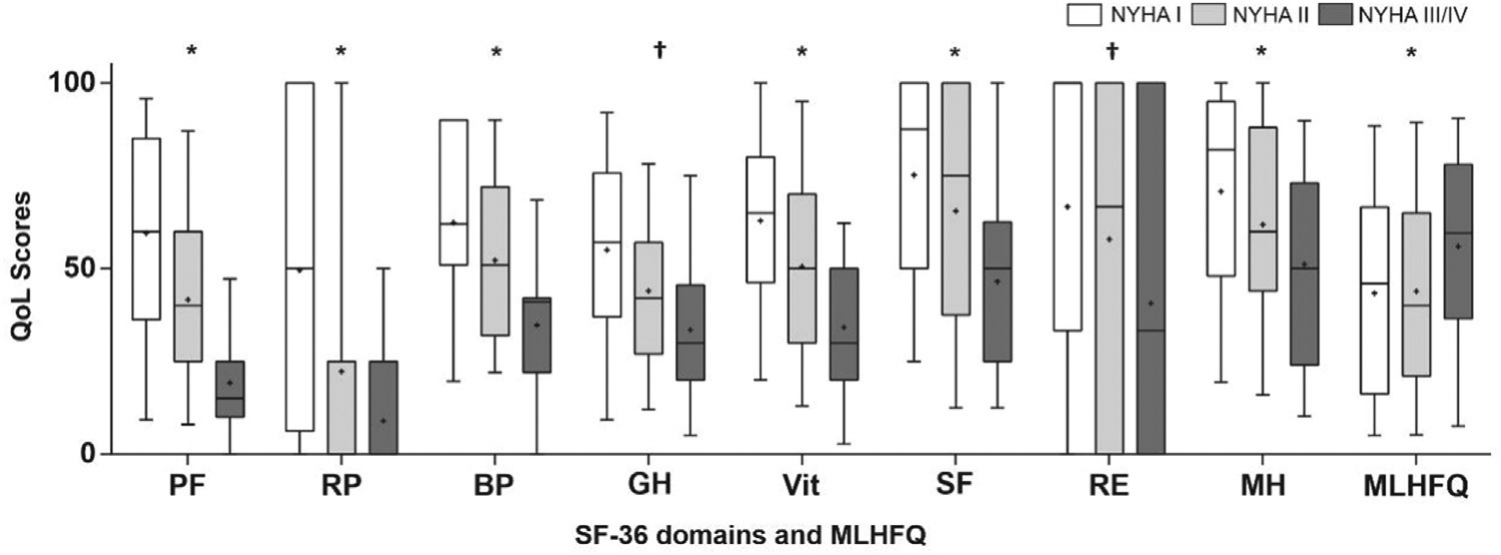
**Quality of life stratified by NYHA functional classification.** Quality of life according to SF-36 domains and MLHFQ scores stratified by NYHA functional classification. QoL, Quality of Life; PF, Physical Functioning; RP, Role Physical; BP, Body Pain; GH, General Health; Vit, Vitality; SF Social Functioning; RE, Role Emotional; MH, Mental Health; MLHFQ, Minnesota Living with Heart Failure Questionnaire; SF-36, Short Form Health Survey. * p < 0.05 in NYHA I vs. NYHA II, NYHA I vs. NYHA III/IV and NYHA II vs. NYHA III/IV † p < 0.05 in NYHA I vs. NYHA III/IV and NYHA II vs. NYHA III/IV.

The median MLHFQ score was 49.5 [IQR 21.0, 69.0], presenting a significant difference among functional classifications (p < 0.05). The BAI was 9.0 [3.0, 21.0], and the BDI was 12.0 [6.0, 22.0]. Questionnaire results are depicted in Appendix 2. Figure 1 shows the SF-36 domains (lower scores indicate worse QoL), and MLHFQ median score (lower scores indicate better QoL) according to NYHA functional classifications, which visually demonstrates a directly proportional relationship between QoL and NYHA. BAI and BDI scores did not present significant differences stratified by NYHA functional classification, but more than 30% of the sample was characterized by moderate-severe anxiety/depression symptoms.

**Fig. 2:**
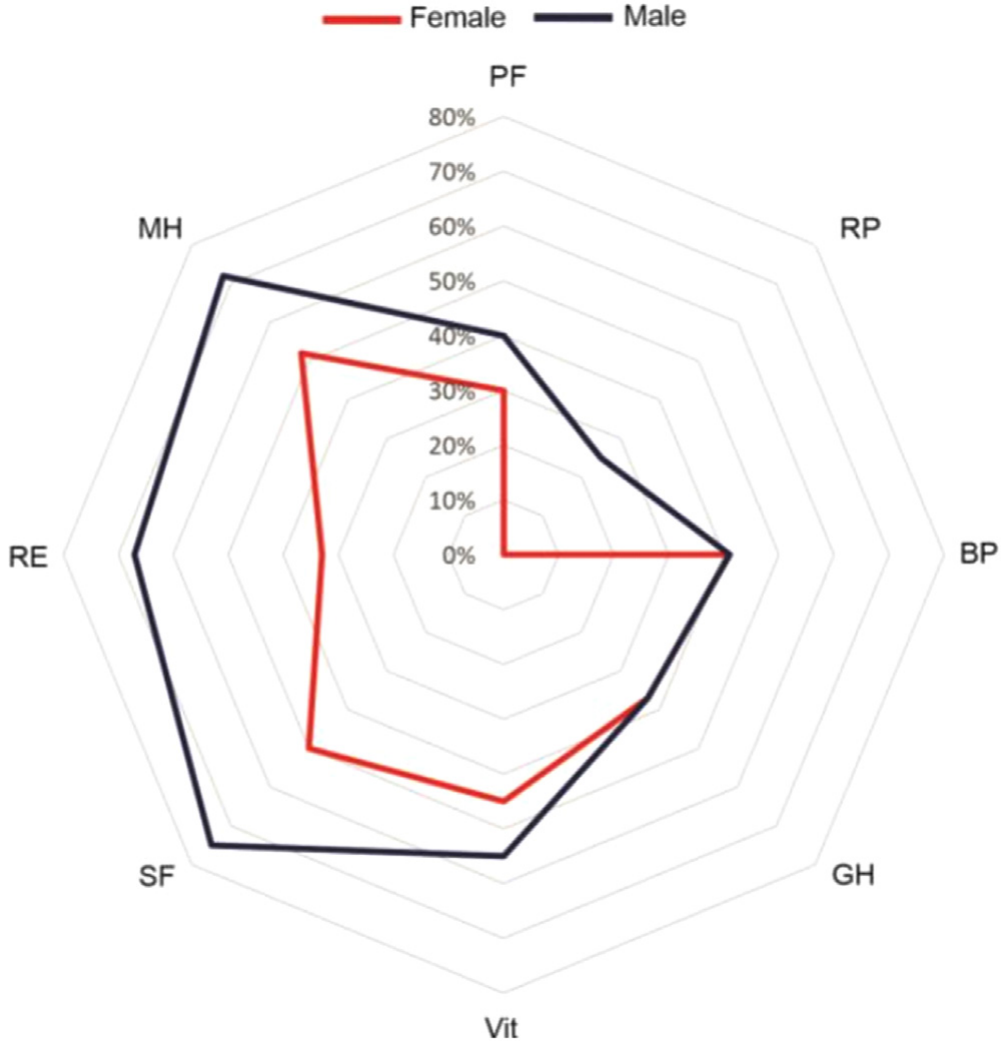
**SF-36 domains according to sex.** Radar chart plot of SF-36 domains according to gender. Axes for domains (PF, Physical Functioning; RP, Role Physical; BP, Body Pain; GH, General Health; Vit, Vitality; SF, Social Functioning; RE, Role Emotional; MH, Mental Health) are equally scaled from 0 to 100. Higher values indicate a better QoL.

Comparing questionnaires between female and male patients, there was a difference in unadjusted analysis in MLHFQ (54.0 [31.0, 73.0] vs. 43.0 [16.0, 66.7] p = 0.01), BAI (15.0 [5.0, 25.0] vs. 6.0 [1.0, 19.0] p < 0.01) and in four SF-36 domains (Physical Functioning p < 0.01, Physical Role p = 0.04, Social Functioning p = 0.02 and Mental Health p < 0.01). The differences were not maintained in the multiple linear regression model, which suggests a strong influence of BAI and BDI scores (p < 0.001, adjusted r^2^ = 0.5), reflecting women's poor mental health. Figure 2 depicts SF-36 domains by gender.

### Costs

When compared by functional classification, a patient classified as NYHA I spent US$ 215.16 ± 238.36 in 6 months, as NYHA II spent US$ 295.68 ± 399.05, and as NYHA III/IV US$ 667.31 ± 1012.57 (Table 2). The medication total cost was similar between NYHA I and NYHA III/IV (US$ 123.94 vs. US$ 135.08), but it corresponded to 57% of the total treatment cost in NYHA I patients, while in NYHA III/IV, it corresponded to 20%. A different pattern is evident in lab/exam costs, which represented almost 30% of the costs in NYHA I, and 74% in NYHA III/IV (US$ 63.26 vs. US$ 491.05).

**Table 2:** Six-month outpatient costs (US$) according to NYHA functional classification.

NYHA	N	Medical appointment	Nurse appointment	Nutritionist appointment	Structure	Medication	Lab and exams	Transportation	Total per patient US$
I	47	7.17 ± 4.72	0.88 ± 1.64	0.29 ± 1.23	5.29 ± 3.40	123.94 ± 131.87	63.26 ± 132.82	14.30 ± 61.35	215.16 ± 238.36
II	89	7.15 ± 6.14	1.31 ± 2.58	0.27 ± 1.32	5.49 ± 4.99	138.46 ± 160.09	137.18 ± 376.96	5.79 ± 9.07	295.68 ± 399.05
III/IV	62	10.84 ± 8.58	3.00 ± 4.64	‒	8.63 ± 6.34	135.08 ± 154.62	491.05 ± 964.68	18.69 ± 70.63	667.31 ± 1012.57

Data are summarized as mean ± standard deviation.

Values are expressed in US dollars.

**Fig. 3:**
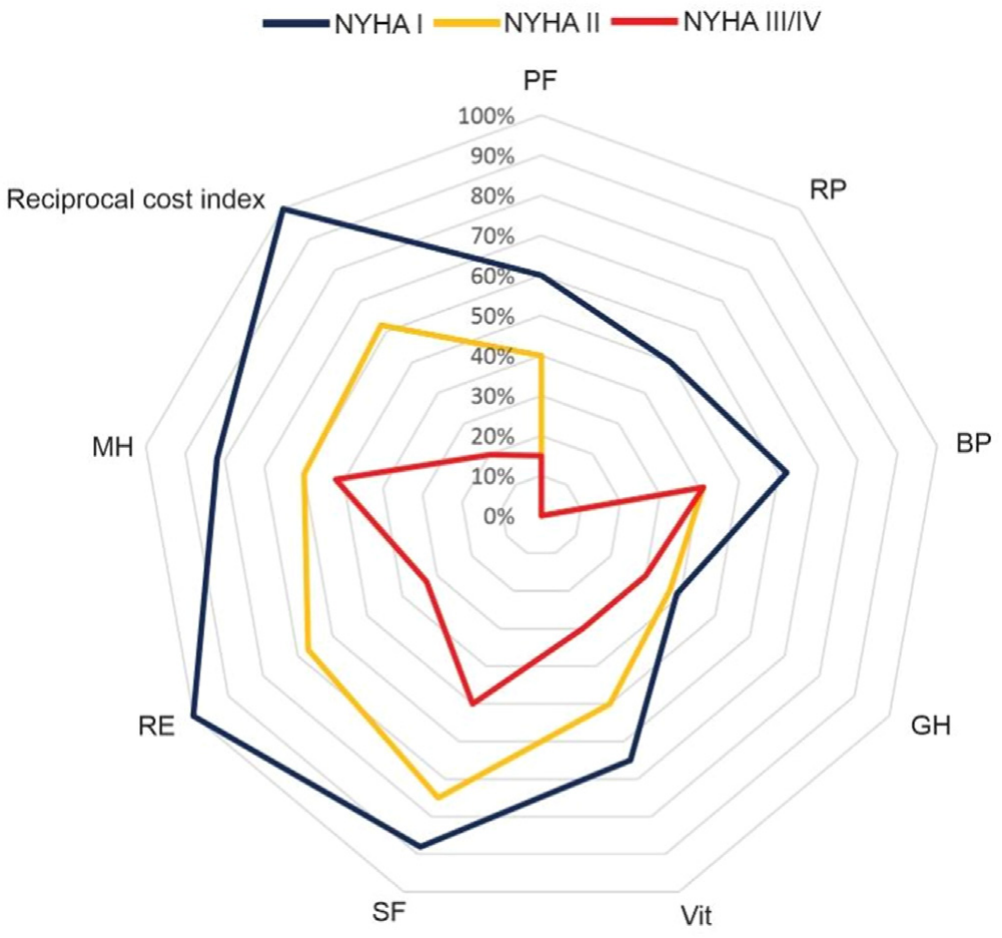
**Quality of life and costs according to NYHA functional classification.** Radar chart plot of SF-36 domains and costs according to NYHA functional classification. Axes for domains (PF, Physical Functioning; RP, Role Physical; BP, Body Pain; GH, General Health; Vit, Vitality; SF, Social Functioning; RE, Role Emotional; MH, Mental Health) as well as the reciprocal cost axis are equally scaled from 0 to 100. One hundred percent means the lowest cost. Higher values indicate a better QoL.

Figure 3 depicts SF-36 quality of life domains and costs according to NYHA functional classification. The differences among functional classifications are clear.

## Discussion

This is a pioneering study that describes QoL and costs according to functional classification in HF outpatients, quantifying the impact that more severe symptoms have on QoL and medical attention, including frequent visits and lab tests, which increases the treatment cost by approximately three times compared with less symptomatic patients. The evaluation of costs, outcomes, and QoL measures in an integrated way as carried out in this research is a fundamental step in bringing the value-based principles in HF care to a particular national context.

Monitoring QoL is essential to achieve better outcomes,^7^ since poor QoL has already been associated with a higher risk of death and hospitalization.^24^ It is starting to be valued in large trials.^25^ Four ICHOM HF standard sets directly involve patients' QoL: symptom control, daily living activities, independence, and psychosocial health.^9^ In this study, more than 50% of the patients had poor QoL, leading us to think that there truly are missing areas in their assistance, such as PROMs’ utilization. The direct association between poorer QoL and higher NYHA classification measured in this study is logical but has not yet been reported.

QoL also includes anxiety and depression evaluations, which frequently do not receive proper attention during follow-up. Our results showed that more than 30% of the patients had anxiety/depression symptoms, which seemed to be worse in women (BAI 15.0 [5.0,25.0] vs. 6.0 [1.0,19.0], p < 0.01). There is evidence in the literature affirming that patients with HF are substantially more affected by mental health issues than the general population and are considered to be factors that are worse in the NYHA classification,^26^ increasing hospitalization rates and mortality.^27^ Studies comparing genders are still missing; there is a difference in the pathophysiology of HF;^28^ however, it does not explain the emergence of anxiety and depression symptoms.^29^

Our cost analysis showed increasing treatment costs according to functional classification, mainly due to spending on exams in more symptomatic patients, NYHA III/IV spent 74% of the budget on labs/exams, while NYHA I spent eight times less. Treatment differences among NYHA classifications must be considered for the establishment of value-payment strategies that may consider the level of specialized services that assist highly complex patients and the outcomes that are being achieved. Payment reform is needed to prioritize the quality of the service instead of the quantity.

With this study, the authors were able to demonstrate the relationship between QoL symptoms and costs. Applying the instruments developed in this study to establish a continuous cycle of PROMs and cost monitoring is an important step for migration to a service based on value. Maddox et al. pointed out that a gap from VBHC models in HF is the mistake of not listening to the patient's experiences, which may be solved by the PROM measurement process and by better patient interactivity with clinicians using, for example, telemedicine strategies that were consolidated during the COVID-19 pandemic.^13^ The adoption of digital care pathways is a key point in reducing long-term costs and improving outcomes.^30^

In Brazil, the current national health program, Sistema Único de Saúde (SUS), has universality, integrality, and equity as principles, being organized according to demographic areas and aiming for a longitudinal and humanized follow-up of patients (a step to Integrated Practice Units ‒ IPU). However, a better-structured flowchart that integrates primary health care and specialized care is still missing, and a mindset change remains a major challenge. Similar examples with the same difficulties around the world can be used as a basis to improve our system, starting with pilot studies such as this.^31^^,^^32^

### Limitations

This study contains a few limitations. The first is the limited sample of patients. The authors are reporting data from 3 HF outpatient services from 2 Brazilian states, which have a good support network for patients when compared to other states. Although these States gather some HF etiological and population variety, expanding the research to other centers will contribute to the achievement of a more representative result. For the cost data, all the analyses reported used the time and resource consumption based on real-world data from each center, but the variable lab/exams were monetarily parametrized using the financial databases from Hospital 1. The authors strongly encourage future studies to use financial datasets from each center.

Another challenge was the time taken to complete all questionnaires, which took approximately 40‒50 minutes, and a significant number of patients refused to join the study due to the time required to answer the questions. In addition, there may have been memory bias and some difficulty in understanding SF-36 questions considering the low educational level of the population.

## Conclusions

Value-based care is important to improve the sustainability of healthcare systems worldwide; however, its dissemination in middle-income countries and universal systems is only slowly starting. In this study, the authors were able to demonstrate that in outpatients with HF in Brazil, QoL worsens, and costs increase in the higher HF functional classification. Additionally, women seem to have worse scores in QoL, anxiety, and depression symptoms. Further studies are needed to generate accurate information to drive proactive care actions that can result in better outcomes and lower costs in the future.

## Funding

The authors would like to thank funding institutions. This work was supported by the 10.13039/501100011644Coordenação de Aperfeiçoamento de Pessoal de Nível Superior (CAPES) [grant number 001]; 10.13039/501100011644Hospital de Clínicas de Porto Alegre and Financiamento e Incentivo à Pesquisa (HCPA/FIPE) [grant number 2018-0442]; 10.13039/501100011644Instituto de Avaliação de Tecnologia em Saúde (IATS) [grant number 17/2551-0000515-5].

## Declaration of Competing Interest

The authors declare no conflicts of interest.

## Appendix Supplementary materials

Image, application 1
