# Supplementary material for: Value-based health care in heart failure: Quality of life and cost analysis
Source: Clinics (Sao Paulo). 2023 Oct 29;78:100294. doi: 10.1016/j.clinsp.2023.100294 (PMC10630105; doi:10.1016/j.clinsp.2023.100294)
Supplement: Supplementary file 1 [file mmc1.doc]

**CLINICS-D-23-00206_Supplementary Material**

**Appendix 1** PROM application instructions.

| Every center must absolutely respect the following rules of the questionnaire application, always applied in a private room. |
| --- |
| - There is freedom regarding the application of the questionnaires; they can be self-administered or read by the researcher. |
| - Whenever possible (literate patient with acceptable cognitive level), the questionnaires should be read and answered by the patients themselves. |
| - In case of doubts, the researcher can only reread the misunderstood sentence, without substituting words or giving different intonation to the text; that is, without explaining the sentence in other words. |
| - In cases in which the researcher will be responsible for reading the questionnaire, the sentences must be read exactly as written, always in the same tone of voice. |
| - Any clarification requested should be answered as “You must answer what you understand from the sentence: (the sentence in question must be reread exactly as written)”. |
| - When the patients answer the questionnaire by themselves, they will be forbidden from receiving help from companions. |

**Appendix 2** Quality of life, anxiety, and depression median scores of included patients stratified by NYHA classification.

|  | **Overall** | **NYHA I** | **NYHA II** | **NYHA III/IV** |
| --- | --- | --- | --- | --- |
| N | 198 | 56 | 92 | 50 |
| MLHFQ ‒ median [IQR] | 49.5 [21.0, 69.0] | 46.0 [16.7, 65.5] | 40.0 [21.0, 62.0] | 59.5 [37.7, 76.5]^a,b,c^ |
| Good – (%) | 62 (31.3) | 32 (57.1) | 29 (31.5) | 1 (2.0) |
| Moderate – (%) | 33 (16.7) | 10 (17.9) | 17 (18.5) | 6 (12.0) |
| Poor – (%) | 103 (52.0) | 14 (25.0) | 46 (50.0) | 43 (86.0) |
| Beck Anxiety Inventory – median [IQR] | 9.0 [3.0, 21.0] | 11.0 [3.0, 20.2] | 8.0 [1.0, 19.0] | 10.0 [3.0, 24.0] |
| Minimal | 102 (51.8) | 27 (48.2) | 51 (55.4) | 24 (49.0) |
| Mild | 34 (17.3) | 12 (21.4) | 16 (17.4) | 6 (12.2) |
| Moderate | 37 (19.8) | 10 (17.9) | 14 (15.2) | 13 (26.5) |
| Severe | 24 (12.1) | 7 (12.5) | 11 (12.0) | 6 (12.2) |
| Beck Depression Inventory – median [IQR] | 12.0 [6.0, 22.0] | 11.0 [6.0, 19.0] | 10. [5.0, 21.5] | 14.0 [9.0, 29.0] |
| Minimal | 80 (40.8) | 22 (39.3) | 45 (49.5) | 13 (26.5) |
| Mild | 52 (26.5) | 19 (33.9) | 17 (18.7) | 16 (32.7) |
| Moderate | 36 (18.4) | 9 (16.1) | 18 (19.8) | 9 (18.4) |
| Severe | 28 (14.3) | 6 (10.7) | 11 (12.1) | 11 (22.4) |
| SF-36 |  |  |  |  |
| Physical Functioning | 35.0 [20.0, 60.0] | 60.0 [38.7, 85.0] | 37.5 [23.7, 60.0] | 15.0 [10.0, 25.0]^b,c^ |
| Role Physical | 0.0 [0.0, 50.0] | 50.0 [18.7, 100.0] | 0.0 [0.0, 25.0] | 0.0 [0.0, 18.7]^a,b,c^ |
| Bodily Pain | 51.0 [31.0, 72.0] | 62.0 [51.0, 90.0] | 51.0 [32.0, 66.0] | 41.0 [22.0, 42.0]^a,b,b^ |
| General Health | 45.0 [25.0, 57.0] | 57.0 [37.0, 73.2] | 42.0 [27.0, 57.0] | 30.0 [20.0, 45.0]^a,b,c^ |
| Vitality | 50.0 [30.0, 70.0] | 65.0 [48.7, 80.0] | 50.0 [30.0, 66.2] | 30.0 [21.2, 50.0]^a,b,c^ |
| Social Functioning | 62.5 [37.5, 100.0] | 87.5 [50.0, 100.0] | 75.0 [37.5, 100.0] | 50.0 [25.0, 62.5]^b,c^ |
| Role Emotional | 66.6 [0.0, 99.9] | 99.9 [33.3, 99.9] | 66.6 [0.0, 99.9] | 33.3 [0.0, 99.9]^a,b,c^ |
| Mental Health | 64.0 [40.0, 88.0] | 82.0 [48.0, 93.0] | 60.0 [44.0, 85.0] | 50.0 [25.0, 72.0]^a,b,c^ |

Variable distributions are reported as n (%) unless otherwise specified. IQR means Interquartile Range.

MLHFQ, Minnesota Living with Heart Failure Questionnaire; NYHA, New York Heart Association; SF-36, Short Form Health Survey.

^a^ = p < 0.05 (NYHA I vs. NYHA II); ^b^ = p < 0.05 (NYHA I vs. NYHA III/IV), ^c^ = p < 0.05 (NYHA II vs. NYHA III/IV)
